# A Narrative Review of Zinc Deficiency-Associated Anemia

**DOI:** 10.3390/hematolrep18040060

**Published:** 2026-08-21

**Authors:** Karina Basmajian, Aren Dermarderosian, Ryan Jeffrey Thoreson, Joshua Adams, Camron Farjami, Austin Yang, Elham Akhtari, Soha Araji, Ziad Khan, Siamak Saadat, Sarkis Arabian, Christina Tansy, Gregory Knutzen, Mojtaba Akhtari

**Affiliations:** 1 Department of Internal Medicine, Arrowhead Regional Medical Center, Colton, CA 92324, USA; 2 University of California, Davis, School of Medicine, Sacramento, CA 95817, USA; 3 Southern Methodist University, Dallas, TX 75205, USA; 4 California University of Science of Medicine, Colton, CA 92324, USA; 5 Palos Verdes Peninsula High School, Rolling Hills Estates, CA 90274, USA; 6 University of California, Berkeley, Berkeley, CA 94720, USA; 7 Department of Traditional Medicine, School of Persian Medicine, Iran University of Medical Sciences, Tehran 1449614535, Iran; 8 Department of Pharmacy, Sharp Healthcare, San Diego, CA 92110, USA; 9 Department of Medicine, Division of Hematology/Oncology, Arrowhead Regional Medical Center, Colton, CA 92324, USA; 10 Department of Medicine, Division of Pulmonology/Critical Care, Arrowhead Regional Medical Center, Colton, CA 92324, USA; 11 Department of Medicine, Division of Transplantation, Cellular Therapy, and Hematological Malignancies, Loma Linda University School of Medicine, Loma Linda University Medical Center, Loma Linda, CA 92354, USA; makhtari@llu.edu

**Keywords:** zinc deficiency, anemia, zinc deficiency-associated anemia, refractory anemia, zinc supplementation

## Abstract

Zinc is an essential micronutrient involved in many physiological processes in the human body, including immune system regulation, cellular survival, neurologic function, and erythropoiesis. Zinc deficiency can result in a wide range of clinical manifestations, including impaired immune function, dermatologic findings, and decreased growth. Anemia has been reported as a hematologic manifestation of zinc deficiency and may be underrecognized in clinical practice. This narrative review examines the pathophysiology of zinc deficiency-associated anemia, highlighting zinc’s role in erythropoiesis, heme biosynthesis, and red blood cell stability. It also outlines populations at increased risk for zinc deficiency, including patients with chronic kidney disease, sickle cell disease, malabsorptive disorders, chronic proton pump inhibitor use, and older adults. Emerging evidence suggests that zinc deficiency-associated anemia may represent a potentially modifiable contributor to anemia in selected at-risk populations, although the strength of evidence varies across clinical settings and study designs. Assessment of zinc status may be considered in patients with unexplained or refractory anemia and additional risk factors for deficiency. Further prospective studies are needed to clarify the role of zinc deficiency in anemia and define appropriate screening, supplementation, and monitoring strategies.

## 1. Introduction

Zinc deficiency is common worldwide and may result from inadequate dietary intake, impaired intestinal absorption, increased losses, or inflammation-driven redistribution that lowers circulating and bioavailable zinc despite normal or near-normal total body stores [1,2,3]. Its clinical manifestations include impaired immune function, dermatologic findings, delayed wound healing, and growth retardation in children [1]. A less widely recognized consequence is its impact on red blood cell production: experimental and clinical data suggest that zinc deficiency can impair heme synthesis, disrupt erythroid differentiation, and increase red blood cell fragility [4,5,6]. These changes may collectively produce or worsen anemia [4,5,6]. In clinical practice, zinc deficiency-associated anemia is often overlooked because anemia is frequently multifactorial and zinc deficiency commonly coexists with iron deficiency, chronic inflammation, chronic kidney disease, copper deficiency, and other micronutrient deficiencies [7,8,9]. The presence of these overlapping conditions can make it difficult to determine the specific contribution of zinc deficiency to anemia [7,8,9].

Recognizing zinc deficiency as a contributor to anemia is especially important in patients with unexplained or refractory anemia, in whom standard evaluation and iron repletion may not fully correct hemoglobin levels [10]. Several populations carry heightened risk for zinc deficiency and zinc deficiency-associated anemia, including individuals with sickle cell disease, pregnancy, chronic kidney disease, malabsorption syndromes and bariatric surgery, long-term proton pump inhibitor use, older adults, and selected genetic disorders affecting zinc transport [10,11,12,13,14]. In many of these populations, observational and interventional studies suggest that zinc repletion may improve hemoglobin and other hematologic indices [10,15,16]. This raises the possibility that zinc deficiency represents a modifiable contributor to anemia that is currently underappreciated in clinical practice [10].

This narrative review examines the evidence on zinc deficiency-associated anemia, covering the pathophysiology of zinc homeostasis and erythropoiesis, clinical manifestations, at-risk populations, diagnostic challenges, and current evidence for management. Our aim is to improve recognition of zinc deficiency as a potential cause or cofactor in anemia and to highlight opportunities for targeted screening and supplementation that may benefit patient outcomes.

## 2. Methods

This narrative review was conducted using literature identified through searches of PubMed and Google Scholar. Search terms included “zinc deficiency anemia,” “zinc and erythropoiesis,” “zinc deficiency and hemoglobin,” “zinc supplementation anemia,” “zinc deficiency chronic kidney disease,” “zinc deficiency sickle cell disease,” “zinc deficiency pregnancy,” and related terms. Additional articles were identified through review of reference lists from relevant publications. The literature was selected based on relevance to the pathophysiology, clinical manifestations, diagnosis, and management of zinc deficiency-associated anemia, including peer-reviewed original studies, review articles, clinical trials, and observational studies. Given the limited available literature in this field, selected conference abstracts and preliminary reports were included when they provided clinically relevant data not otherwise available in the peer-reviewed publications.

## 3. Pathophysiology of Zinc Deficiency-Associated Anemia

### 3.1. Overview

Anemia is one of the most prevalent hematological disorders worldwide, affecting approximately 1 in 4 people globally [17]. The most common cause of anemia is iron deficiency [18]. However, zinc deficiency can also contribute to anemia, particularly in specific populations such as the elderly and patients with chronic kidney disease [10,16]. Zinc deficiency-associated anemia likely reflects two overlapping biological states: absolute zinc deficiency and functional zinc deficiency [19,20]. Absolute deficiency arises from reduced intake, impaired absorption, or increased losses, while functional deficiency results from inflammation-driven redistribution that lowers circulating and bioavailable zinc despite adequate intake [1,21]. During the acute-phase response, inflammatory cytokines alter zinc trafficking, producing hypozincemia that may mimic true deficiency on laboratory testing [2,21]. Reduced bioavailable zinc impairs erythropoiesis through disruption of heme biosynthesis, erythroid differentiation, and red blood cell oxidative stability [2,22].

### 3.2. Absorption and Metabolism

**Figure 1:**
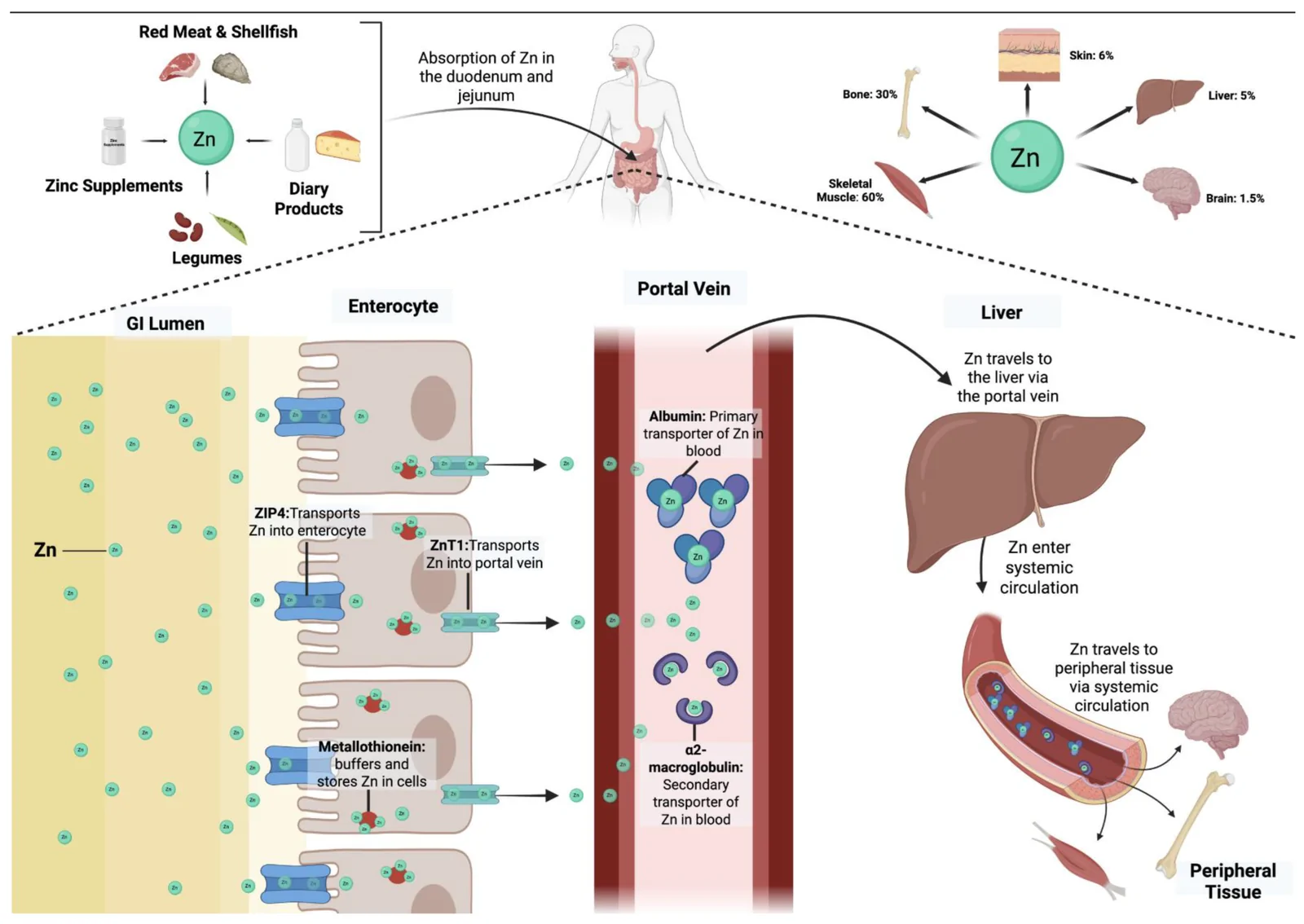
Intestinal absorption and systemic distribution of zinc. Dietary zinc is primarily absorbed in the duodenum and jejunum via the apical transporter ZIP4. Within enterocytes, zinc is buffered by metallothionein or transported into the portal circulation via ZnT1. In plasma, zinc is predominantly bound to albumin, with α2-macroglobulin as a secondary carrier, and is delivered to the liver before distribution to peripheral tissues, including muscle, bone, skin, liver, and brain.

Zinc homeostasis is regulated by two transporter families [23]. ZIP transporters (SLC39) increase cytosolic zinc by importing zinc into cells, while ZnT transporters (SLC30) reduce cytosolic zinc by exporting it or sequestering it into intracellular compartments [23]. In the intestine, zinc absorption occurs primarily in the proximal small bowel [19]. ZIP4 (SLC39A4) functions as the principal apical transporter responsible for zinc uptake into enterocytes [24]. Disruption of ZIP4 results in severe systemic zinc deficiency, as observed in acrodermatitis enteropathica [24,25]. After uptake, zinc exits the enterocyte via the basolateral transporter ZnT1 (SLC30A1), allowing zinc to enter systemic circulation [23]. Disruption of these transport pathways contributes to zinc deficiency and may impair erythropoiesis [25] (Figure 1).

### 3.3. Zinc in Bone Marrow Function and Erythropoiesis

Reduced zinc availability impairs erythropoiesis through several mechanisms [6]. Zinc participates in enzymatic processes required for erythroid development, including heme biosynthesis [6]. Experimental models demonstrate that cellular zinc deficiency reduces porphyrin synthesis and hemoglobin production during erythroid differentiation even in the presence of adequate iron [6]. Zinc also regulates erythropoiesis through zinc-finger transcription factors [4]. The transcription factor GATA-1 contains zinc-finger domains and is essential for erythroid maturation and survival [4]. Disruption of this regulatory pathway impairs terminal erythroid differentiation [4].

### 3.4. Zinc and Oxidative Stress

Zinc deficiency also compromises red blood cell membrane stability through increased oxidative stress [6]. Zinc functions as a cofactor for Cu/Zn superoxide dismutase, an antioxidant enzyme that protects erythrocyte membranes from lipid peroxidation [26]. Clinical studies in zinc-deficient hemodialysis patients demonstrate that zinc supplementation can reduce oxidative membrane damage and improve red blood cell fragility, supporting a role for oxidative stress in zinc deficiency-related anemia [5,27].

### 3.5. Zinc, Inflammation, and the Hepcidin Axis

Systemic inflammation significantly alters zinc physiology [22]. Zinc is largely albumin-bound in plasma, and acute-phase responses lower circulating zinc concentrations through redistribution rather than true depletion [22]. Interleukin-6 (IL-6) induces hepatic expression of metallothionein and the zinc transporter ZIP14 (SLC39A14) [2]. ZIP14 promotes hepatic zinc uptake, while metallothionein binds intracellular zinc and functions as a buffering reservoir during inflammatory stress [2,3]. This inflammatory redistribution parallels mechanisms seen in iron metabolism [28]. IL-6 stimulates hepatic production of hepcidin, which limits iron export and restricts iron availability for erythropoiesis [28]. Unlike iron, zinc homeostasis lacks a comparable endocrine regulator, and hypozincemia instead results primarily from transporter-mediated redistribution and intracellular sequestration [22]. In addition to inflammatory signaling, hepcidin expression is also regulated by erythropoietic activity through erythroferrone (ERFE), an erythroid-derived hormone that suppresses hepcidin production to enhance iron availability for erythropoiesis [29]. Recent clinical data have demonstrated associations between ERFE levels and markers of iron status, highlighting the contribution of erythropoietic signaling to iron regulation [29]. These findings further emphasize the complex interplay between inflammation, erythropoietic signaling, and iron metabolism in the pathophysiology of anemia [28,29].

### 3.6. Iron and Zinc Interactions

Interactions with other micronutrients further complicate the pathophysiology of zinc deficiency [30]. Iron and zinc share partially overlapping intestinal absorption pathways, and high doses of non-heme iron can reduce zinc absorption [30]. Copper deficiency represents an important interaction [31]. Excess zinc induces enterocyte metallothionein expression, which preferentially binds copper [31]. Copper trapped in enterocytes is lost during epithelial turnover, potentially leading to secondary copper deficiency [31]. Copper deficiency itself may present with anemia, neutropenia, and bone marrow abnormalities [31,32].

## 4. Congenital Zinc Deficiency Syndromes

Although typically acquired, zinc deficiency-associated anemia may also occur in genetic disorders that result in severe zinc deficiency [33]. The two primary congenital zinc deficiency syndromes are acrodermatitis enteropathica (AE) and transient neonatal zinc deficiency (TNZD) [14,33]. Both conditions classically present with periorificial and acral dermatitis, alopecia, and diarrhea [33].

AE is an autosomal recessive disorder caused by mutations in SLC39A4, which encodes the ZIP4 zinc intestinal transporter, leading to impaired intestinal zinc absorption [33]. On the other hand, TNZD is caused by mutations in the SLC30A2 gene, which codes for the zinc transporter ZnT2, leading to reduced zinc transport into the breast milk and deficiency in exclusively breastfed infants [34,35]. Clinical manifestations are indistinguishable from AE; however, TNZD resolves once an infant is weaned from breast milk and receives adequate dietary zinc, whereas AE requires lifelong supplementation [33,36].

In congenital zinc deficiency syndromes, anemia has been reported but is not traditionally emphasized as a defining complication [37]. In a pediatric review of patients with AE, decreased hemoglobin (mean hemoglobin 9.2 g/dL) was reported in a substantial number of cases, suggesting that severe inherited zinc deficiency can be associated with clinically significant anemia [38]. These conditions demonstrate that severe zinc deficiency, regardless of whether acquired or inherited, can impair erythropoiesis and contribute to anemia [33,38].

## 5. Clinical Manifestations of Zinc Deficiency-Associated Anemia

Zinc deficiency-associated anemia typically presents with nonspecific symptoms of anemia, including fatigue, weakness, pallor, and decreased exercise tolerance [39,40]. These symptoms reflect zinc’s role in erythroid development, including heme biosynthesis [4]. One study focusing on middle-aged and elderly adults reported that low zinc concentrations were associated with normocytic anemia, suggesting that zinc deficiency may contribute to anemia in this population [41]. However, the hematologic characteristics of zinc deficiency-associated anemia have not been well defined, as zinc deficiency frequently coexists with chronic disease and other micronutrient deficiencies, most commonly iron deficiency [39,42]. Other laboratory features, including red blood cell morphology, may therefore be influenced by these overlapping conditions [39,42]. Populations at higher risk of zinc deficiency include individuals with sickle cell disease, pregnancy, chronic kidney disease, and malabsorption disorders [42]. Recognition of zinc deficiency-associated anemia is challenging, as symptoms and laboratory findings are largely nonspecific and may overlap with other causes of anemia [42,43]. Given this variable clinical presentation, maintaining clinical suspicion for zinc deficiency-associated anemia may be particularly important in at-risk populations [42,43].

## 6. Zinc Deficiency in Specific Populations

### 6.1. Sickle Cell Disease

Patients with sickle cell disease (SCD) have been shown to exhibit deficiencies in multiple vitamins and minerals, including zinc [44]. This has been attributed to increased metabolic demand and consumption, chronic hemolysis, and increased urinary excretion due to impaired renal concentration and hypoxanthinuria, despite having a normal diet [44]. Zinc deficiency in adults with SCD was first reported in 1975, with one study reporting decreased zinc concentrations in the plasma, erythrocytes, and hair in approximately 60–70% of patients compared to controls [45,46]. Subsequent pediatric cohorts have consistently demonstrated a high prevalence of zinc deficiency in SCD [47,48]. In a Nigerian cohort, 67% of children with SCD were zinc deficient compared with 34% of matched controls, while in the ZIPS trial approximately 60–64% of young children had low serum zinc concentrations [47,48]. Although adult prevalence data remain more limited than pediatric data, multiple studies have demonstrated significantly lower serum zinc levels in adults with SCD compared with healthy controls [44,49,50]. These findings highlight the need for additional studies evaluating the prevalence and clinical implications of zinc deficiency in adult SCD populations [51].

Anemia in SCD is primarily driven by chronic hemolysis; however, coexisting zinc deficiency may further contribute to impaired erythropoiesis and altered heme biosynthesis [44]. In addition to hemolysis, oxidative stress plays a major role in the pathophysiology of SCD and contributes to erythrocyte membrane damage and reduced red blood cell survival [52]. Zinc possesses antioxidant properties through its role as a cofactor for Cu/Zn superoxide dismutase, stabilization of cellular membranes, promotion of metallothionein synthesis, and inhibition of NADPH oxidase activity [53]. Zinc deficiency may therefore contribute to oxidative injury and impaired erythrocyte membrane stability, potentially exacerbating hemolysis and worsening anemia in some patients with SCD [44].

Several clinical studies have evaluated zinc supplementation in both adults and children with SCD and reported improvements in hematologic and oxidative stress parameters [54,55]. Clinical studies evaluating hematologic responses to zinc supplementation across populations at risk for zinc deficiency-associated anemia are summarized in Table 1. In a randomized placebo-controlled trial, adults receiving 25 mg of zinc orally three times daily for 3 months showed significant increases in red blood cell count, hemoglobin, and hematocrit compared with placebo, and there was also a noted decrease in oxidation products [54]. Pediatric studies have also demonstrated similar improvements with zinc supplementation [55]. In a single-arm prospective study, children with SCD who received daily oral zinc sulfate for 12 months exhibited significant increases in hemoglobin, hematocrit, fetal hemoglobin, and antioxidant activity with a significant decrease in ferritin levels, suggesting reduced inflammation and improved erythropoietic function [55]. Although anemia in SCD is primarily hemolytic, these findings suggest that correction of zinc deficiency may reduce oxidative stress and inflammation, potentially contributing to improved erythrocyte stability and hematologic parameters in selected patients [44]. However, the available interventional data remain limited, and larger prospective studies are needed to better define the role of zinc supplementation in this population [44,54,55].

Given the high prevalence of zinc deficiency in patients with SCD, assessment of zinc status may be considered in selected patients, particularly those with unexplained worsening anemia, evidence of nutritional deficiency, or increased hemolytic complications [44].

### 6.2. Pregnancy

Pregnant women are at increased risk for zinc deficiency due to increased maternal and fetal zinc requirements, although dietary factors, baseline nutritional status, and other clinical conditions may also contribute [11]. In a study of 400 pregnant women in Iran, 49% were found to have zinc deficiency [11]. Surveys revealed that dietary intake, maternal age, and supplemental iron were associated with zinc deficiency [11]. Dietary patterns in this cohort were characterized by higher fiber intake and lower consumption of zinc-rich foods, factors that may reduce zinc bioavailability [11]. Women ages 20–30 years were more likely to be affected, potentially reflecting increased zinc requirements during pregnancy [11].

In addition to dietary factors, iron supplementation has been shown to influence zinc absorption [56]. A study of pregnant Peruvian women compared supplementation with iron and folate alone versus iron, folate, and zinc [56]. Results demonstrated that fasting iron supplementation reduced zinc absorption by up to 50% compared with combined zinc supplementation [56]. These findings suggest that high-dose iron supplementation may impair zinc absorption under certain conditions, although the clinical significance of this interaction may vary depending on dietary intake, formulation, and timing of supplementation [56].

In addition to maternal anemia, zinc deficiency has been associated with adverse pregnancy outcomes including low birth weight and preterm delivery [57]. However, the role of routine zinc screening and supplementation during pregnancy remains incompletely defined and warrants further investigation [57].

### 6.3. Chronic Kidney Disease

Chronic kidney disease (CKD) affects 1 in 10 people globally and is often associated with micronutrient deficiencies, including zinc [58]. Several mechanisms contribute to zinc deficiency in this population, including dietary protein restriction, decreased caloric intake, intestinal malabsorption, hyperuricemia, decreased kidney reabsorption and urinary wasting, increased fecal excretion, and hemodialysis [58]. Multiple studies and meta-analyses have demonstrated significantly lower zinc levels in patients with CKD compared with healthy controls [59]. A cohort study conducted in Switzerland revealed that lower zinc levels were associated with greater declines in the glomerular filtration rate (GFR) [59].

As discussed previously, zinc is essential for erythropoiesis and antioxidant defense [60,61]. Therefore, zinc deficiency may further impair erythroid maturation and heme biosynthesis, reducing hemoglobinization and limiting effective red blood cell production in CKD, a condition where oxidative stress and chronic inflammation are already pronounced [6,62]. While anemia in CKD is largely multifactorial with causes including iron deficiency, chronic inflammation, and impaired erythropoietin production, zinc deficiency may represent a potentially modifiable contributor to anemia in selected CKD patients [7,8].

In hemodialysis patients, low zinc status has been associated with erythropoiesis-stimulating agent (ESA) hyporesponsiveness, which may contribute to persistent anemia [63]. Oral zinc supplementation has been shown to reduce the erythropoietin responsiveness index in some anemic patients in this population, suggesting that zinc status may influence ESA responsiveness in selected individuals [63]. Limited interventional data also suggest that zinc supplementation may improve hematologic parameters in CKD patients with zinc deficiency [64]. In a small retrospective cohort study of CKD patients with both zinc deficiency and chronic anemia, zinc supplementation was associated with improvement in hemoglobin levels, with resolution of anemia observed in 35% of patients over 1 year [64].

Although these findings suggest that zinc deficiency may contribute to anemia in some CKD patients, the available evidence remains limited and is derived largely from observational studies and retrospective analyses. Additional prospective studies and randomized controlled trials are needed to determine whether zinc supplementation provides clinically meaningful benefits and to establish appropriate screening, dosing, and monitoring strategies in CKD populations [64]. Assessment of zinc status may be considered in selected CKD patients with unexplained or refractory anemia, particularly when additional risk factors for zinc deficiency are present [64].

### 6.4. Older Adults

Zinc deficiency is also commonly reported in older adults [15]. It may result from lack of nutritional zinc intake, poor mastication, and complex social situations that may limit nutritional adequacy [15]. Other contributors include decreased gastric acid secretion and reduced absorption in the small intestine, the primary site of zinc absorption [65]. These factors may increase the risk of zinc deficiency and may contribute to anemia in susceptible individuals [15,65].

In a cohort of 285 adults over the age of 65 who resided in a care facility, 72% had zinc deficiency, and 32% were anemic [10]. Notably, individuals with zinc deficiency were five times more likely to also have anemia [10]. In a separate study, 59 patients who were over age 70 with chronic anemia received zinc supplementation [10]. After 12 months of treatment, the average hemoglobin increased from 10.1 g/dL to 11.01 g/dL, and the average serum zinc levels increased from 52.8 μg/dL to 70.8 μg/dL [10]. In addition, 39% of these patients were no longer anemic [10]. While these findings suggest that zinc deficiency may be an underrecognized contributor to anemia in some older adults, the available evidence remains limited and additional prospective studies are needed to better define the role of zinc supplementation in this population [10]. Given the increased risk of zinc deficiency among older adults, assessment of zinc status may be considered in selected patients with unexplained or refractory anemia, particularly when additional risk factors for zinc deficiency are present [10].

### 6.5. Proton Pump Inhibitor Use

Proton pump inhibitors (PPIs) are widely prescribed medications that function by blocking the H^+^/K^+^ ATPase enzyme in gastric parietal cells [66]. This results in increased gastric pH, which can disrupt zinc absorption by not allowing it to dissolve into its ionic form, which is required for absorption [12,13,16]. As a result, chronic PPI use has been proposed as a potential risk factor for zinc deficiency and may contribute to zinc deficiency-associated anemia in susceptible individuals [16].

Management of zinc deficiency in patients who are on chronic PPIs may include zinc supplementation and reassessment of the ongoing indication for PPI therapy [16]. However, discontinuation of PPI therapy is not always clinically feasible or appropriate. In one study, patients with zinc deficiency who were receiving chronic PPI therapy underwent treatment with zinc supplementation and simultaneously discontinued PPI therapy [10]. Results after 12 months of treatment demonstrated significant increases in hemoglobin, with average hemoglobin increasing from 9.6 g/dL to 11.3 g/dL, and the average serum zinc level increasing from 50.8 μg/dL to 61.5 μg/dL [10]. In addition, 48% of the patients achieved resolution of anemia after 12 months [10]. However, because zinc supplementation and PPI discontinuation were implemented concurrently, the relative contribution of each intervention to the observed hematologic improvement could not be determined [10].

These findings suggest that it may be reasonable to assess zinc status in patients on long-term PPI therapy, particularly patients who also have anemia of unknown cause [10,16]. When zinc deficiency is detected, clinicians should consider zinc supplementation and also reassess the ongoing need for PPI therapy when clinically appropriate [10,16].

### 6.6. Malabsorption Syndromes and Bariatric Surgery

Zinc is primarily absorbed in the small intestine, with absorption being highest at the jejunum followed by the duodenum [65]. Therefore, certain conditions that affect the small intestine are associated with an increased risk for zinc deficiency; this includes disorders such as celiac disease and inflammatory bowel disease (IBD) as well as patients who have undergone bariatric surgery [67,68,69]. Studies have reported that zinc deficiency has prevalence rates ranging from 40 to 54% in IBD, 59.4% in celiac disease, and 26.1–42.5% in patients 1 year after undergoing bariatric surgery [67,68,69,70,71,72,73].

Anemia in these populations is common; however, it is typically multifactorial, with contributors including chronic inflammation, gastrointestinal blood loss, and other micronutrient deficiencies, including iron, copper, vitamin B12, and folate [73,74]. Given the high prevalence of zinc deficiency in these populations, inadequate zinc status may represent an additional contributor to anemia in selected patients [71,73]. As discussed previously, zinc plays an essential role in erythropoiesis, and impaired zinc absorption may further contribute to ineffective red blood cell production in susceptible individuals [6,74].

Limited evidence suggests that zinc supplementation may improve hematologic parameters in zinc-deficient patients in the post-bariatric surgery population [75]. In a study presented at the American Society of Hematology Annual Meeting in 2024, mean hemoglobin increased from 9.6 g/dL at baseline to 12.2 g/dL at 12 months following oral zinc replacement, with anemia resolution noted in 50% of patients [75]. However, these findings are derived from abstract-level data and should be considered preliminary pending publication of larger prospective studies [75]. Further research is needed to clarify the contribution of zinc deficiency to anemia in malabsorptive disorders and to determine the role of zinc supplementation, optimal screening strategies, and replacement protocols in these populations [75]. Assessment of zinc status may be considered in patients with malabsorptive disorders or prior bariatric surgery who present with unexplained or refractory anemia, particularly when other causes have been excluded.

## 7. Zinc Supplementation

### 7.1. Formulations and Clinical Considerations

The most common over-the-counter (OTC) zinc supplement formulations include zinc gluconate, zinc citrate, zinc picolinate, zinc acetate, and zinc sulfate, each selected for distinct physicochemical characteristics and clinical applications as they differ in bioavailability, gastrointestinal (GI) tolerability, and elemental zinc content [76]. Zinc gluconate and zinc citrate are among the most commonly used formulations due to their good tolerability and absorption [76]. Zinc picolinate, a chelated compound, has favorable solubility and tolerability, though its higher absorption potential may increase the risk of copper deficiency with prolonged use [77]. Zinc acetate is highly soluble and well absorbed, while zinc sulfate contains a high proportion of elemental zinc, but it is less commonly utilized due to its greater likelihood of causing GI irritation [76,77].

The choice of formulation in clinical practice is typically determined based on patient tolerance and side effect profile [76]. For systemic repletion in zinc deficiency-associated anemia, formulations with good bioavailability and tolerability are generally preferred [77,78]. Regardless of the formulation used, prolonged zinc supplementation should be monitored due to the risk of secondary copper deficiency [78].

Zinc supplementation may also interact with the absorption of other micronutrients [56]. A small physiologic study of 47 pregnant women in Peru demonstrated that prenatal iron supplementation was associated with reduced zinc absorption under fasting conditions [56]. Clinicians should therefore be aware of potential iron–zinc interactions when both supplements are prescribed [56]. However, evidence-based recommendations regarding the timing or separation of zinc and iron supplementation remain limited.

### 7.2. Dosing and Management of Zinc Deficiency-Associated Anemia:

Zinc deficiency-associated anemia is typically treated with approximately 50 mg of elemental zinc once daily, most often administered as zinc sulfate 220 mg [64]. This dosing strategy is supported by clinical studies demonstrating improvement in hemoglobin levels over time [64]. Hemoglobin responses tend to increase gradually, with average rises of 0.6 g/dL at 1 month, 0.9 g/dL at 3 months, 1.5 g/dL at 6 months, and 1.6 g/dL at 12 months, and about 42% of patients achieving full resolution of anemia by 1 year [64].

However, dosing should be individualized based on the underlying cause and severity of zinc deficiency, patient characteristics, comorbidities, and tolerability, as optimal dosing strategies for specific clinical populations have not been established [10,64].

More broadly, management of zinc deficiency in the literature generally involves supplementation at 2–5 times the recommended daily allowance (RDA), which corresponds to roughly 16–55 mg of elemental zinc per day in adults [79]. Treatment duration varies, but many clinicians continue supplementation for 3–6 months before reassessing zinc status and hematologic parameters [79]. Monitoring of hemoglobin and serum zinc concentrations during supplementation may help assess treatment response, although standardized monitoring intervals have not been established [10]. Because copper deficiency can itself cause hematologic abnormalities including anemia, neutropenia, and marrow dysplasia, the risk of zinc-induced copper deficiency is an important consideration during prolonged zinc therapy [80,81]. Consideration should therefore be given to monitoring copper levels during treatment, particularly in patients requiring long-term therapy [78]. Excess zinc can impair copper absorption through induction of enterocyte metallothionein, which binds copper and reduces its systemic availability [31]. However, evidence-based recommendations regarding copper monitoring intervals and thresholds for copper replacement remain limited [78].

Specific treatment targets, including target hemoglobin responses and serum zinc concentrations during supplementation, have not been established [10]. Furthermore, optimal zinc dosing strategies for pediatric and pregnant populations with zinc deficiency-associated anemia remain unclear, as studies in these populations are limited [55,56].

## 8. Diagnosis & Clinical Management

### 8.1. Diagnostic Evaluation of Zinc Deficiency-Associated Anemia

The diagnosis of zinc deficiency-associated anemia requires integration of laboratory biomarkers with clinical evaluation, particularly in patients with unexplained or refractory anemia [20]. Because zinc is classified as a Type II nutrient, zinc deficiency results in nonspecific biochemical and clinical manifestations rather than a single pathognomonic indicator [20,82]. Diagnosis is further complicated by the tight homeostatic regulation of zinc within the human body, which maintains circulating zinc concentrations within a narrow range [83]. As a result, clinical symptoms often appear only after prolonged deficiency overwhelms the body’s homeostatic mechanisms [82]. Zinc deficiency should be considered in patients with anemia that is unexplained, refractory to therapy, or occurring in patients who are at higher risk for zinc deficiency [42].

Serum or plasma zinc is the most commonly used biomarker and the only indicator with widely accepted cutoff values for diagnosing zinc deficiency [84]. According to the International Zinc Nutrition Consultative Group (IZiNCG), serum zinc concentrations below approximately 57 µg/dL in children (<10 years), 59 µg/dL in women, and 61 µg/dL in men in non-fasting afternoon samples are suggestive of zinc deficiency [85]. Higher cutoffs are used for morning fasting samples (approximately 70 µg/dL for women and 74 µg/dL for men) because serum zinc concentrations exhibit diurnal variation and decline following food intake [84]. However, serum zinc has important limitations, as circulating zinc only accounts for <0.1% of the body’s total zinc, with most of it highly conserved in the muscles and bones [20,42]. Serum zinc concentrations are influenced by multiple physiological and external factors, such as diurnal variation, recent meal consumption, medication use, hormonal fluctuations, pregnancy, serum albumin concentrations, and specimen hemolysis, which can confound results [42,82,85]. Therefore, borderline serum zinc concentrations should be interpreted cautiously and in conjunction with clinical risk factors, inflammatory states, and evaluation for other causes of anemia. When the clinical suspicion for zinc deficiency remains high despite an inconclusive zinc concentration, repeat measurement under standardized conditions may be considered [42,82,85].

Systemic inflammation can further complicate the interpretation of zinc status, particularly in patients with anemia of chronic disease [42]. During inflammatory states, circulating zinc is redistributed to the liver due to cytokine-induced hepatic metallothionein synthesis, which binds circulating zinc within liver cells, leading to reduced circulating zinc levels independent of true deficiency [42]. Given this, measurement of inflammatory markers such as C-reactive protein (CRP) and α1-acid glycoprotein can aid in the interpretation of serum zinc levels [85].

Although alternative biomarkers have been investigated, their clinical utility remains limited [82]. These include hair, nail, urinary, and erythrocyte zinc measurements [82]. Urinary zinc excretion may reflect changes in zinc intake; however, further research is required as data are limited for women and children [86].

Erythrocyte zinc concentration has been proposed as a more stable biomarker due to its intracellular distribution, providing a more reliable assessment during acute inflammatory states [87]. However, current evidence suggests that erythrocyte zinc is not a reliable biomarker of zinc status due to its inconsistent responsiveness to dietary zinc intake [82]. These limitations underscore that the diagnosis of zinc deficiency-associated anemia relies primarily on clinical context and should not be based on laboratory findings alone [82].

Importantly, the relationship between zinc deficiency and anemia remains complex, as observed associations may be influenced by multiple overlapping factors [10]. Many populations at risk for zinc deficiency also have concurrent contributors to anemia, including iron deficiency, chronic inflammation, CKD, malabsorption, and other micronutrient deficiencies [10]. Furthermore, low circulating zinc concentrations in inflammatory states may reflect altered zinc distribution rather than isolated zinc depletion [42]. Therefore, low serum zinc concentrations should not be interpreted as definitive evidence that zinc deficiency is the sole cause of anemia; rather, zinc deficiency should be considered a potential contributing factor within a broader evaluation of anemia [42].

Given these diagnostic limitations and the absence of standardized diagnostic criteria or validated algorithms specifically for zinc deficiency-associated anemia, evaluation should incorporate assessment of zinc status, interpretation of results within clinical context, consideration of inflammatory states, and evaluation for other causes of anemia [42]. Practical considerations for the evaluation and management of zinc deficiency-associated anemia are summarized in Table 2. 

### 8.2. When to Suspect Zinc Deficiency-Associated Anemia

Zinc deficiency should be considered in patients with unexplained or refractory anemia [10]. Although clinical manifestations are often nonspecific, the presence of dermatologic findings such as periorificial dermatitis and alopecia, impaired growth, immune dysfunction, delayed wound healing, or gastrointestinal symptoms such as diarrhea and anorexia in a patient with anemia should raise suspicion for zinc deficiency [85,88,89]. Zinc deficiency should be considered in patients with known risk factors, including those discussed in the preceding sections of this paper [42]. These include individuals with chronic diseases, physiologic states with increased zinc demand, malabsorption disorders, dietary insufficiencies, older adults, and certain genetic disorders (Table 3) [42]. Clinical suspicion of zinc deficiency-associated anemia should guide laboratory testing and workup, as early recognition could help prevent progression of anemia and its associated complications [84].

## 9. Monitoring Parameters

Monitoring parameters for zinc supplementation in anemia include serum zinc and hemoglobin levels, which should be monitored at 3, 6, and 12 months during zinc supplementation for anemia [10]. Additional laboratory markers that may be followed include red blood cell count (RBC), mean corpuscular volume (MCV), and ferritin [10]. Serum zinc levels can be influenced by inflammatory status; however, current methods to adjust zinc concentrations for inflammation are still being refined and are primarily used in research and population-level work rather than routine clinical practice [90,91,92]. Serum zinc also has several practical limitations as a biomarker: it responds less to zinc obtained from food than to supplements administered between meals, shows considerable interindividual variability, and is influenced by recent food intake, time of day, inflammation, and certain drugs and hormones [42]. Serum zinc deficiency is generally defined as <60 μg/dL, although the Office of Dietary Supplements suggests sex specific cut-offs of <70 μg/dL for women and <74 μg/dL for men [85]. Copper should also be monitored while on long-term zinc supplementation as prolonged zinc supplementation can induce copper deficiency, which may lead to cytopenias and bone marrow changes that closely resemble myelodysplastic syndromes (e.g., dysplastic precursors, vacuolization, and ring sideroblasts) [80].

## 10. Safety Recommendations

As discussed previously, long-term high-dose zinc supplementation can lead to copper deficiency, which may cause hematologic complications such as anemia, neutropenia, or pancytopenia [81]. These effects have been reported with chronic zinc intake of 100–300 mg/day [81]. Acute zinc toxicity has also been linked to thrombocytopenia, anemia, and hyperamylasemia [93].

## 11. Conclusions

Zinc deficiency-associated anemia has not received sufficient attention in public health and routine clinical practice; it remains an underrecognized clinical entity with a complex pathophysiology. Multiple populations, including older adults, patients with chronic kidney disease, chronic proton pump inhibitor users, and post-bariatric surgery patients, may be at increased risk for zinc deficiency that can contribute to anemia. Zinc deficiency should be considered in patients with unexplained or refractory anemia, particularly when risk factors for deficiency are present. Emerging evidence suggests that zinc supplementation may improve hemoglobin levels in selected zinc-deficient populations [10,75]. Further research and prospective studies are needed to clarify the role of zinc deficiency in anemia, establish optimal screening strategies, and define evidence-based supplementation and monitoring recommendations.

## Figures and Tables

**Table 1:** Clinical studies evaluating hematologic parameters following zinc supplementation in populations at risk for zinc deficiency-associated anemia. Abbreviations: PO, orally; TID, three times daily; mo, months; ↑, indicates an increase in the specified parameter; +, indicates coexistence of clinical characteristics; QD, once daily.

Population	Study Design	Sample Size (n)	Intervention	Findings	Limitations
Adults with SCD	Randomized- control trial	36	Zinc 25 mg PO TID for 3 mo	↑ Hgb	Short follow-up duration
Pediatric patients with SCD	Single-arm prospective	60	Zinc sulfate PO daily for 12 mo	↑ Hgb	No control group
Adults with CKD3-5 + zinc deficiency + chronic anemia	Retrospective cohort	34	Zinc Sulfate 50 mg QD for 12 mo	↑ Hgb, resolution of anemia in 35% of patients	Retrospective design, no control group
Older adults (>70 years) + zinc deficiency + chronic anemia	Retrospective cohort	59	Zinc sulfate 220 mg QD for 12 mo	↑ Hgb, resolution of anemia in 39% of patients	Retrospective design, no control group
Adults on chronic PPI + zinc deficiency + chronic anemia	Retrospective cohort	48	Zinc sulfate 220 mg QD for 12 mo + discontinuation of PPI	↑ Hgb, resolution of anemia in 48% of patients	Retrospective design, no control group
Adults with prior bowel surgery or bariatric surgery or malnutrition + zinc deficiency + chronic anemia	Retrospective cohort	23	Zinc sulfate 220 mg QD for 12 mo	↑ Hgb, resolution of anemia in 50% of patients	Retrospective design, no control group

**Table 2:** Practical considerations for evaluation and management of zinc deficiency-associated anemia.

Clinical Consideration	Practical Approach
When to evaluate for zinc deficiency	Consider assessment of zinc status in patients with unexplained or refractory anemia and risk factors for zinc deficiency (CKD, SCD, malabsorption disorders, chronic PPI use, pregnancy, older age)
Assessment of zinc status	Measure serum zinc concentration as the primary assessment of zinc status. Interpret results in the context of inflammation, fasting status, diurnal variation, and dietary intake.
Evaluation of anemia	Evaluate for other common contributors to anemia, including iron deficiency, chronic inflammation, CKD, and other micronutrient deficiencies, as zinc deficiency-associated anemia frequently occurs in the setting of multiple contributing factors.
Role of inflammatory markers	Consider concurrent assessment of inflammatory markers (e.g., CRP) when interpreting serum zinc concentrations, as inflammation may lower circulating zinc levels through redistribution rather than true depletion.
Zinc supplementation	Consider zinc supplementation in patients with laboratory evidence of or a high clinical suspicion of zinc deficiency. Clinical studies have commonly used doses of approximately 50 mg elemental zinc daily. However, optimal dosing, treatment duration, and target zinc levels remain undefined. Dosing should be individualized based on patient-specific factors, including age, underlying cause and severity of deficiency, pregnancy status, comorbidities, and risk of zinc-related adverse effects, including copper deficiency.
Monitoring and Safety Recommendations	Monitor hematologic response and zinc status during and following supplementation. Consider monitoring copper levels during prolonged or high-dose zinc therapy due to the risk of zinc-induced copper deficiency, which may contribute to anemia. Evidence-based monitoring intervals and copper replacement thresholds have not been established.

**Table 3:** Populations at increased risk for zinc deficiency, with associated risk factors and proposed mechanisms.

Category	Risk Factors	Mechanism
Medication/Supplementation	Phenytoin; tetracyclines/quinolones; iron supplements; penicillamine; ethambutol; diuretics (e.g., chlorothiazide)	Reduced zinc absorption (phenytoin, tetracyclines/quinolones); competitive inhibition of zinc absorption (iron supplements); zinc chelation reducing tissue availability (penicillamine, ethambutol); increased urinary zinc excretion (diuretics)
Dietary	High-phytate diets (cereals, legumes, corn, rice); low-protein diets; low animal-source foods	Low dietary zinc intake; reduced bioavailability due to phytate binding; reduced absorption in low-protein or low animal-source diets
Malabsorption and Gastrointestinal Disorders	IBD (Crohn’s, ulcerative colitis); celiac disease; steatorrhea/fat malabsorption	Intestinal inflammation and zinc loss (IBD); impaired intestinal absorption (celiac disease); formation of insoluble zinc–fat complexes and increased fecal zinc losses (steatorrhea)
Physiological States/Increased Requirements	Pregnancy; lactation; infancy; adolescence	Increased zinc demand due to tissue growth and development; zinc transfer to fetus (pregnancy) or breast milk (lactation); rapid growth increasing zinc requirements (infancy, adolescence)
Chronic Diseases	Chronic liver disease; chronic kidney disease; alcoholism	Impaired zinc metabolism and utilization; increased zinc losses
Older Adults	Low dietary intake; reduced appetite; dental problems; food insecurity	Reduced dietary intake due to lower energy needs; physical limitations affecting food consumption (e.g., swallowing or dental problems)
Genetic Disorders	Acrodermatitis enteropathica (AE); Transient neonatal zinc deficiency (TNZD)	Impaired intestinal zinc absorption (AE); reduced zinc content in breast milk due to maternal transporter defect (TNZD)

## Data Availability

No new data were created or analyzed in this study. Data sharing is not applicable to this article.
